# Preconcentration and Determination of Copper(II) in Water and Tea Infusion Samples Using Hierarchical MnSb_2_O_6_@Fe_3_O_4_ Nanoparticles and Magnetic Solid Phase Extraction–FAAS

**DOI:** 10.1021/acsomega.4c10772

**Published:** 2025-02-26

**Authors:** Dilges Baskin

**Affiliations:** Muradiye Vocational School, Chemistry and Chemical Processing Technologies Department, Van Yuzuncu Yil University, Van 65080, Turkey

## Abstract

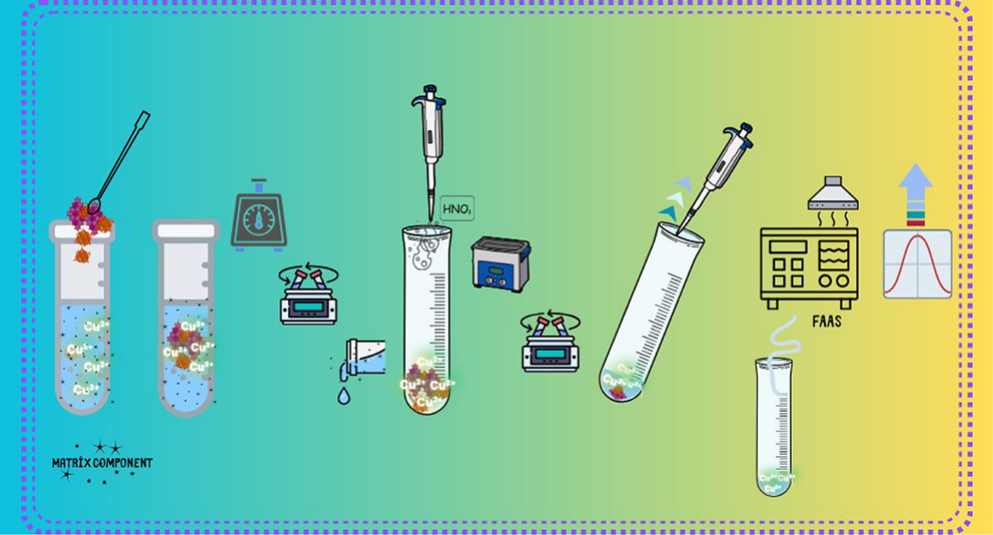

Heavy metal pollution poses a significant threat to living organisms and requires continuous monitoring of the environmental samples. In this study, novel hierarchical MnSb_2_O_6_@Fe_3_O_4_ nanoparticles were synthesized and used as adsorbents in magnetic solid-phase extraction (MSPE) of Cu(II). The strong magnetic properties of these nanoparticles enabled rapid and efficient separation from complex matrices, simplifying preconcentration and ensuring high adsorption efficiency. By integration of MSPE with flame atomic absorption spectrometry (FAAS), matrix effects were reduced, detection limits improved, and the cost-effectiveness and simplicity of FAAS were leveraged for Cu(II) analysis in complex samples. The optimized parameters for the MSPE-FAAS method included pH, stirring time and type, eluent volume and type, and adsorbent amount, achieving a correlation coefficient of 0.9991, a limit of detection of 2.1 ng·mL^–1^ and a linear range of 10.0–200 ng·mL^–1^. The developed method enhanced FAAS sensitivity by 48-fold and was successfully applied to wastewater, tap water, and apple tea samples, achieving recoveries of 93–102%. The Cu(II) adsorption capacity of MnSb_2_O_6_@Fe_3_O_4_ was determined to be 15.2 mg·g^–1^, demonstrating its high efficiency for heavy metal removal. This methodology highlights a robust and efficient approach for preconcentrating trace metals from diverse and complex matrices, combining the advantages of MSPE and FAAS in a practical, cost-effective system.

## 1 Introduction

Heavy metal pollution poses a significant threat to living organisms and adversely affects the entire food pyramid with a cumulative impact on human metabolism. Various components of heavy metals are found in environmental and food samples.^1^ Some metals are essential for biological processes, but others, even at trace levels, can exhibit toxic effects. For example, copper is a ubiquitous heavy metal crucial for various biological processes.^2^ However, its accumulation in the environment can harm plant, animal, and human health.^3^

Detecting heavy metals from complex matrices such as environmental and food samples presents significant challenges. Conventional laboratory techniques, such as inductively coupled plasma optical emission spectroscopy and mass spectrometry, have been widely used to analyze metal contaminants.^4^ While effective, these methods are time-consuming, require extensive sample preparation, and are costly to implement. Flame atomic absorption spectroscopy (FAAS), on the other hand, is relatively inexpensive, offers simple operation, and is widely used.^5^ However, there is a need for preconcentration techniques in FAAS due to high detection limits and matrix effect problems, particularly when analyzing real samples with complex and varying matrices.^6^ Additionally, FAAS provides reliable and precise single-element analysis, making it particularly suitable for routine applications in environmental and food matrices, where cost-effectiveness and accessibility are critical.

In complex matrices, organic structures, salts, or other metal ions mixed with the FAAS flame can enter the light path and cause interference. The interference effect from complex matrices reduces the sensitivity and accuracy of FAAS.^7^ For example, easily^8^ ionizable elements, such as sodium and potassium, can lead to ionization interferences, and refractory compounds can cause chemical interferences^9^ in FAAS. Research has focused on pretreatment techniques to reduce or eliminate interference effects, and a common one is solid phase extraction (SPE). With the intensification of SPE research, various SPE techniques have been described. Magnetic solid phase extraction (MSPE) is one of them, where the extraction process is facilitated by a magnet.^6^ In the MSPE technique, the separation of the adsorbent from the filtrate is carried out with the help of a magnet, and as a result, the classical SPE procedure is simplified.^10^

The use of magnetic materials offers significant advantages, including rapid and efficient separation, reduction of sample preparation time, and ease of operation. The development of novel magnetic adsorbents, such as Fe_3_O_4_-based materials, has shown great potential in enhancing the extraction and preconcentration of metal ions for subsequent analysis by FAAS.^11^ Studies have demonstrated the successful application of magnetic nanoparticles in SPE to preconcentrate various metal ions, including lead and cadmium, before atomic absorption spectrometry analysis.^12^ In this study, Fe_3_O_4_ was chosen for its strong magnetic properties, which enable efficient handling and separation from complex matrices, as well as its modifiability to improve the adsorption capacity for Cu(II) ions. Many studies on the effective use of Fe_3_O_4_ as an adsorbent are available, which support modification studies of this adsorbent.

This study presents a promising approach for the efficient and selective extraction and detection of Cu(II) ions by using MnSb_2_O_6_@Fe_3_O_4_ (MNP) magnetic nanoparticles as an adsorbent for magnetic solid phase extraction (MSPE) of Cu(II) in FAAS. The novelty of this study lies in the hierarchical design of MnSb_2_O_6_@Fe_3_O_4_, which combines the high adsorption capacity of MnSb_2_O_6_ with the rapid magnetic separation capability of Fe_3_O_4_, offering a highly effective solution for the analysis of trace metals in complex matrices.

## 2 Experimental Section

### 2.1 Materials

The chemicals used in the experiment were carefully selected and purchased from AB Chemical, a trusted supplier in the industry. The purchased chemicals included antimony trichloride (SbCl_3_), iron(III) chloride hexahydrate (FeCl_3_·6H_2_O), iron(II) chloride tetrahydrate (FeCl_2_·4H_2_O), manganese(II) chloride (MnCl_2_), and nitric acid (HNO_3_). Standard stock solutions containing 1000 mg·L^–1^ copper were supplied by High-Purity Standards (Charleston, SC, USA) and were diluted as needed for the experiments. Buffer solutions were prepared using potassium hydrogen phthalate (pH 3–6) from Merck and Tris buffer (pH 7), borax (pH 8–10), disodium hydrogen phosphate (pH 11–12), NaOH, and HCl.

### 2.2 Instrumentation

The experimental setup, designed for precision, involved using ultrapure water supplied by a Merck Millipore Direct-Q 3 UV filter system to dilute calibration and working standard solutions. Absorbance measurements for quantifying Cu were conducted using a flame-type atomic absorption spectrophotometer (Thermo Scientific ICE-3000 Series, USA). A copper hollow cathode lamp (HCL) was the radiation source, operating at 5.0 mA with a spectral bandpass of 0.5 nm. The analytical wavelength used was 324.8 nm for Cu. A stoichiometric flame generated from a mix of acetylene as the fuel gas and air as an oxidant ensured optimal absorption measurements.

### 2.3 Synthesis of MNP (MnSb_2_O_6_@Fe_3_O_4_)

**Figure 1:**
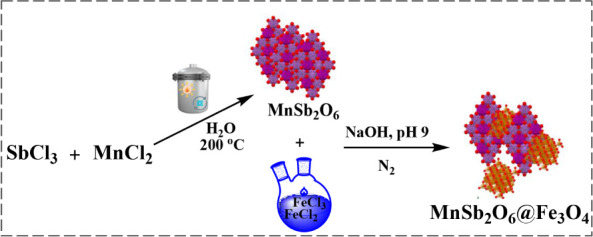
Schematic representation of the synthesis of MnSb_2_O_6_@Fe_3_O_4_.

The synthesis process of MnSb_2_O_6_@Fe_3_O_4_ is illustrated in Figure 1. MnSb_2_O_6_ was first synthesized as a precursor for MnSb_2_O_6_@Fe_3_O_4_. The synthesis of MnSb_2_O_6_ nanoparticles was carried out using a solvothermal method.^13^ 5.6 g of SbCl_3_ (0.02 mmol) and 0.75 g of MnCl_2_·4H_2_O (4 mmol) were mixed in 10 mL of ultrapure water. The mixture was then treated in a Teflon-coated hydrothermal reactor at 200 °C for 3 h. To eliminate the nonparticipating salts from the synthesis, the medium underwent a series of washes with distilled water until reaching a neutral pH. The obtained hierarchical MnSb_2_O_6_ particles were ready for the subsequent synthesis step.

MNP (MnSb_2_O_6_@Fe_3_O_4_) nanoparticle synthesis followed a method described in the literature.^14^ The process began with transferring 0.03 g of FeCl_2_·4H_2_O (0.17 mmol) and 0.09 g of FeCl_3_·6H_2_O (0.34 mmol) into 10 mL of distilled water. To this solution added 0.4 g of previously synthesized MnSb_2_O_6_ (1 mmol) under N_2_. As the solution was mixed for an hour using a mechanical stirrer, 8 M NaOH solution was added dropwise until the pH reached 9. High-speed centrifugation was then performed to obtain nanosized MnSb_2_O_6_@Fe_3_O_4_. The synthesized nanoparticles were thoroughly washed with deionized water and ethanol/acetone to remove the unreacted material.

#### 2.3.1 Characterizations

The X-ray diffractometer (XRD) peaks of the synthesized MnSb_2_O_6_ and MnSb_2_O_6_@Fe_3_O_4_ particles were analyzed using a Rigaku Ultima IV device with Cu Kα radiation in the 2θ range 10–90°. This analytical technique allowed for the determination of the crystal structures of the materials. Additionally, the three-dimensional morphological features of the particles were observed through field emission scanning electron microscopy (FESEM) by using a Zeiss Sigma VP 300 machine. The elemental content of MnSb_2_O_6_ and MnSb_2_O_6_@Fe_3_O_4_ particles was determined by FESEM’s X-ray detector (EDX). Consequently, XRD, FESEM, EDX, and FTIR techniques were used to characterize the particles. In this way, the particles’ structural, morphological, surface, and elemental structures were characterized.

#### 2.3.2 Adsorption Procedure

To evaluate the adsorption performance of hierarchical MnSb_2_O_6_@Fe_3_O_4_ nanoparticles, a batch adsorption method was utilized. In the experiments, 50 mg of the MnSb_2_O_6_@Fe_3_O_4_ adsorbent was added to 200 mL of a Cu(II) solution with an initial concentration of 10 mg·L^–1^ at pH 9. The adsorption process was carried out in a 250 mL erlenmeyer flask, and the mixture was stirred at 300 rpm for 60 min using a mechanical stirrer to ensure thorough interaction between the adsorbent and the analyte ions.

After adsorption, the adsorbent was separated from the solution using a combination of a magnetic field and centrifugation at 6000 rpm to ensure complete removal from the supernatant. The supernatant was then filtered to remove any remaining particulates and analyzed by FAAS to determine the equilibrium concentration of Cu(II) ions in the solution. All experiments were conducted in triplicate to ensure reproducibility and reliability of the results.

The adsorption capacity (*q*_e_) of the MnSb_2_O_6_@Fe_3_O_4_ nanoparticles and the removal efficiency (%) were calculated using the following equations:
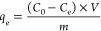
1

Here, *q*_e_ represents the adsorption capacity of the adsorbent in mg·g^–1^, *C*_0_ is the initial concentration of Cu(II) ions (ng·mL^–1^), *C*_e_ is the equilibrium concentration after adsorption (ng·mL^–1^), *V* is the volume of the solution in liters (L), and *m* is the mass of the adsorbent in grams (g).

This procedure allowed for a comprehensive evaluation of the adsorbent’s performance under optimized conditions, providing critical insights into its efficiency and selective binding capacity for Cu(II) ions.

#### 2.3.3 Magnetic Solid Phase Extraction Procedure

The MSPE-FAAS extraction procedure begins with the preparation of standard solutions at different concentrations from a 1000 ppm Cu(II)-containing metal ion stock solution. The standard or real sample solution is mixed with a buffer solution, followed by the addition of MNPs. To enhance the interaction between MNPs and the analyte, the optimal mixing method and duration were determined, and the mixing process was conducted under these conditions. Subsequently, the MNPs were centrifuged at 6000 rpm to precipitate at the bottom of the falcon tube. The supernatant was carefully separated from the precipitated particles, while the MNPs were stabilized by using a magnet. To dissolve the analyte adsorbed on the MNPs, a concentrated acid solution was added. To ensure proper dissolution of the analyte in the acidic eluent, the solution was mixed for the optimum duration. The eluent was then carefully collected from the precipitated particles, with the magnet used again to stabilize the particles. Finally, the eluent was ready for absorbance measurement, which was performed under optimized conditions using the FAAS instrument.

To enhance the performance of the proposed MSPE analytical method, parameters such as pH, particle type and amount, eluent type and volume, and mixing conditions were optimized. Under optimal conditions, the performance of the MSPE-FAAS method was comprehensively evaluated. Performance parameters used to assess the precision and accuracy of the method included the limit of detection (LOD), standard deviation (SD), determination coefficient (*R*^2^), and percent relative standard deviation (% RSD), and the adsorption capacity of the adsorbent.^15^

The limit of detection (LOD) represents the lowest concentration of an analyte that can be reliably detected. The standard deviation (SD) measures the dispersion of data points around the mean, reflecting the precision of the method. The determination coefficient (*R*^2^) is a statistical measure that evaluates how well the data fits a regression model. The percent relative standard deviation (%RSD) indicates method precision by expressing the standard deviation as a percentage of the mean value.

For the lowest concentration with a signal-to-noise ratio of 3, seven replicate MSPE-FAAS experiments were conducted. Additionally, to assess the analytical performance of the method, absorbance values of a series of standard solutions were recorded in triplicate by using the MSPE-FAAS method.

#### 2.3.4 Preparation of the Real Samples

The MSPE-FAAS method was rigorously evaluated for sensitivity and reliability through comprehensive recovery studies. Real wastewater and tap water samples were used, and the method was applied to 50-fold diluted solutions of these samples. The wastewater sample was sourced from the VASKİ (Van General Directorate of Water and Sewerage Administration), and the tap water sample was taken directly from the laboratory tap. Apple tea samples were purchased from a local market. 10 g of tea was weighed, brewed with 500 mL of boiled water, and filtered. 100-fold dilution was used for the final recovery samples of tea infusions.

## 3 Results and Discussions

### 3.1 Characterizations of MnSb_2_O_6_@Fe_3_O_4_ (MNP) Particles

**Figure 2:**
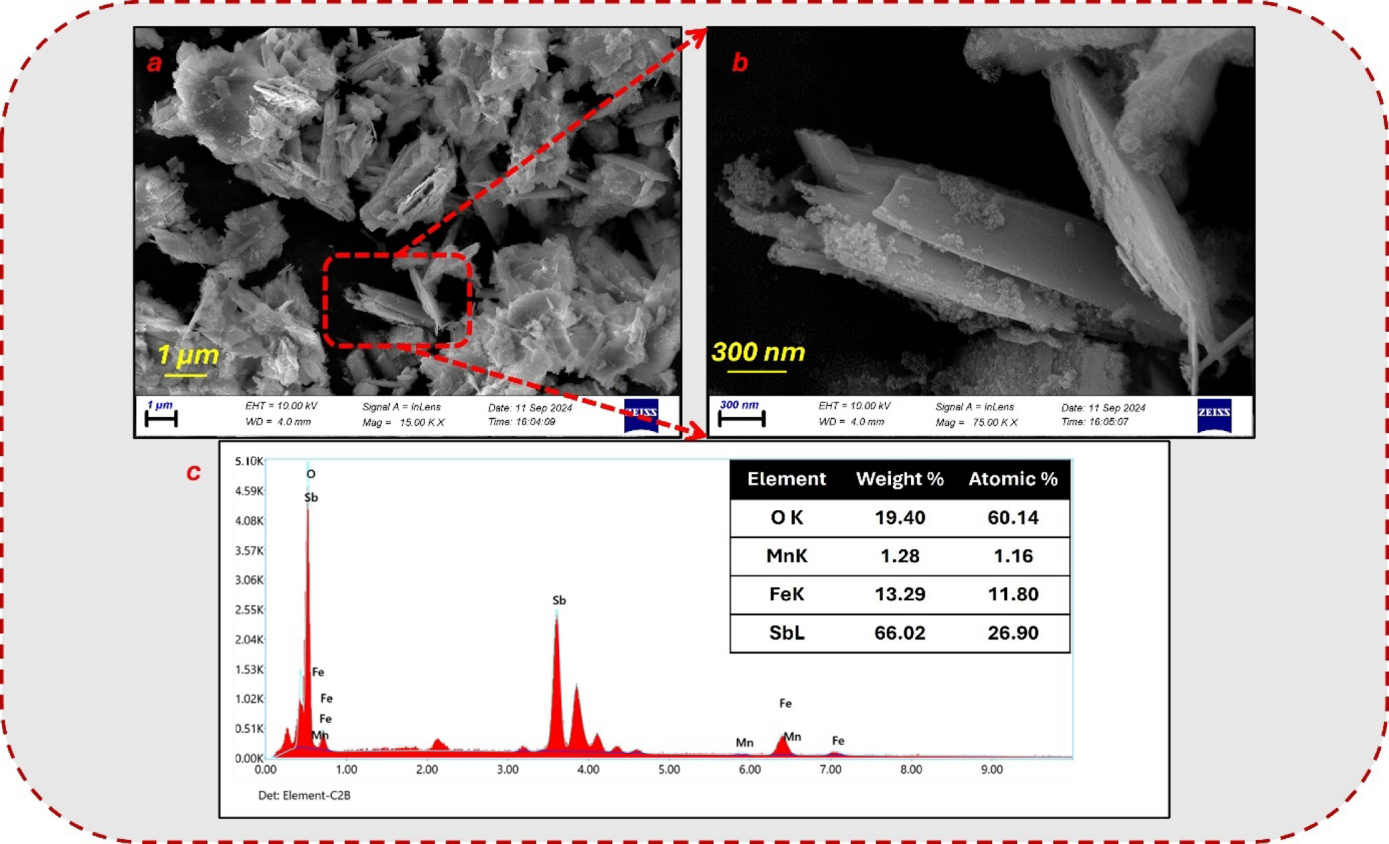
FESEM images and EDX spectrum of MnSb_2_O_6_@Fe_3_O_4_. (a) FESEM image with a scale of 1 μm. (b) FESEM image with a scale of 300 nm. (c) EDX spectrum shows the elemental composition of MnSb_2_O_6_@Fe_3_O_4_.

The hierarchical layered morphological structure of MnSb_2_O_6_@Fe_3_O_4_ was investigated with FESEM images. FESEM is a sophisticated imaging technique widely used to characterize nanoscale material surfaces. The FESEM image in Figure 2a shows the surface image of the material magnified 15,000 times. The material’s surface, which has a layered and bulk structure, is covered with magnetite. The image indicated by Figure 2b presents a detailed surface image of the 3D material, magnified 75,000 times with higher resolution. The particles resemble crystalline structures and have sharp corners. Their morphology shows an anisotropic thin-layered growth trend, and the nanoparticle size ranges from 250 to 400 nm. The elemental structure of MnSb_2_O_6_@Fe_3_O_4_ was investigated using FESEM-EDX spectra, a semiquantitative method. Upon examination of the FESEM-EDX elemental analysis spectrum in Figure 2c and the accompanying table in the inset, it is evident that the structure comprises the elements Sb, O, Fe, and Mn.

Regarding the overall surface morphology , the material’s layered structure may increase the surface area and contribute to adsorption. Interlayer spacings and orientations may also be essential in adsorption mechanisms and kinetics. For example, it has been reported that the layered structure of nanoplates in BiOCl nanoplates increases their surface area and specific morphology, facilitating interaction with adsorbates and improving adsorption properties.^16^

**Figure 3:**
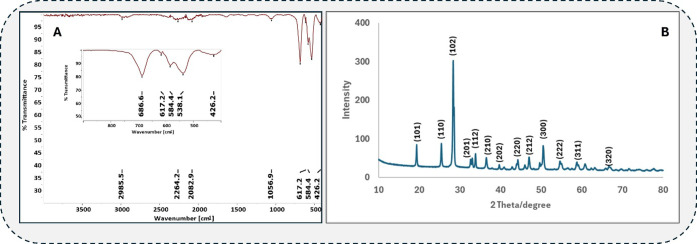
(A). FTIR spectrum of MnSb_2_O_6_@Fe_3_O_4_ and (B). XRD spectrum of the crystallographic structure of MnSb_2_O_6_@Fe_3_O_4_.

Figure 3A shows the FTIR spectrum of the MnSb_2_O_6_@Fe_3_O_4_ hierarchical composite structure. The 400–700 cm^–1^ range absorption bands are often attributed to metal–oxygen bonds’ bending and stretching vibrations.^17^ Accordingly, the peaks at 538 and 584 cm^–1^ are associated with Fe–O stretching vibrations and support the formation of Fe_3_O_4_.^18^ Furthermore, the 617 and 686 cm^–1^ bands correspond to Mn–O or Sb–O bonds and are associated with the MnSb_2_O_6_ structure.^19^ The sharp and distinct diffraction peaks in the XRD spectrum in Figure 3B indicate a high degree of crystallinity. The peaks shown as (102), (110), (201), and (112) confirm^20^ the crystal structure of MnSb_2_O_6_, while the peaks (220), (222), and (311) correspond to the Fe_3_O_4_ structure.^21^ The SEM, SEM-EDX, FTIR, and XRD outputs agree with standard crystallographic data and provide strong evidence for successfully integrating MnSb_2_O_6_ and Fe_3_O_4_ into a composite material with the desired structural and chemical properties.

### 3.2 Analytical Method Optimization

In the MSPE-FAAS method, univariate optimization was used to increase the signal-to-noise ratio and achieve lower detection limits in FAAS. Univariate optimization enhances analytical method performance by systematically optimizing the parameters.^22^ Optimizing one variable at a time allows for a more targeted improvement in signal quality. This can lead to a reduction in detection limits and an increase in the accuracy of the analytical method.^23^ This methodological strategy is crucial to achieving lower detection limits by fine-tuning specific parameters to maximize the signal-to-noise ratio and improve the efficiency of analytical methods such as FAAS.^22^

#### 3.2.1 Optimization of pH

Given the inevitable changes in the behavior and structure of metal ions in different ionic environments, pH is a critical parameter to optimize metal ion extraction. The interaction of hydroxyl or hydronium species with metal ions is heavily influenced by the pH of the medium. In high pH regions, metal ions either precipitate or form hydroxyl complexes. The pH also plays a crucial role in determining the solubility and cation valence of metal ions. Moreover, the pH significantly impacts the surface charge or structure of the adsorbent, a key player in the adsorption process, which can either increase or decrease the adsorption.^24^

**Figure 4:**
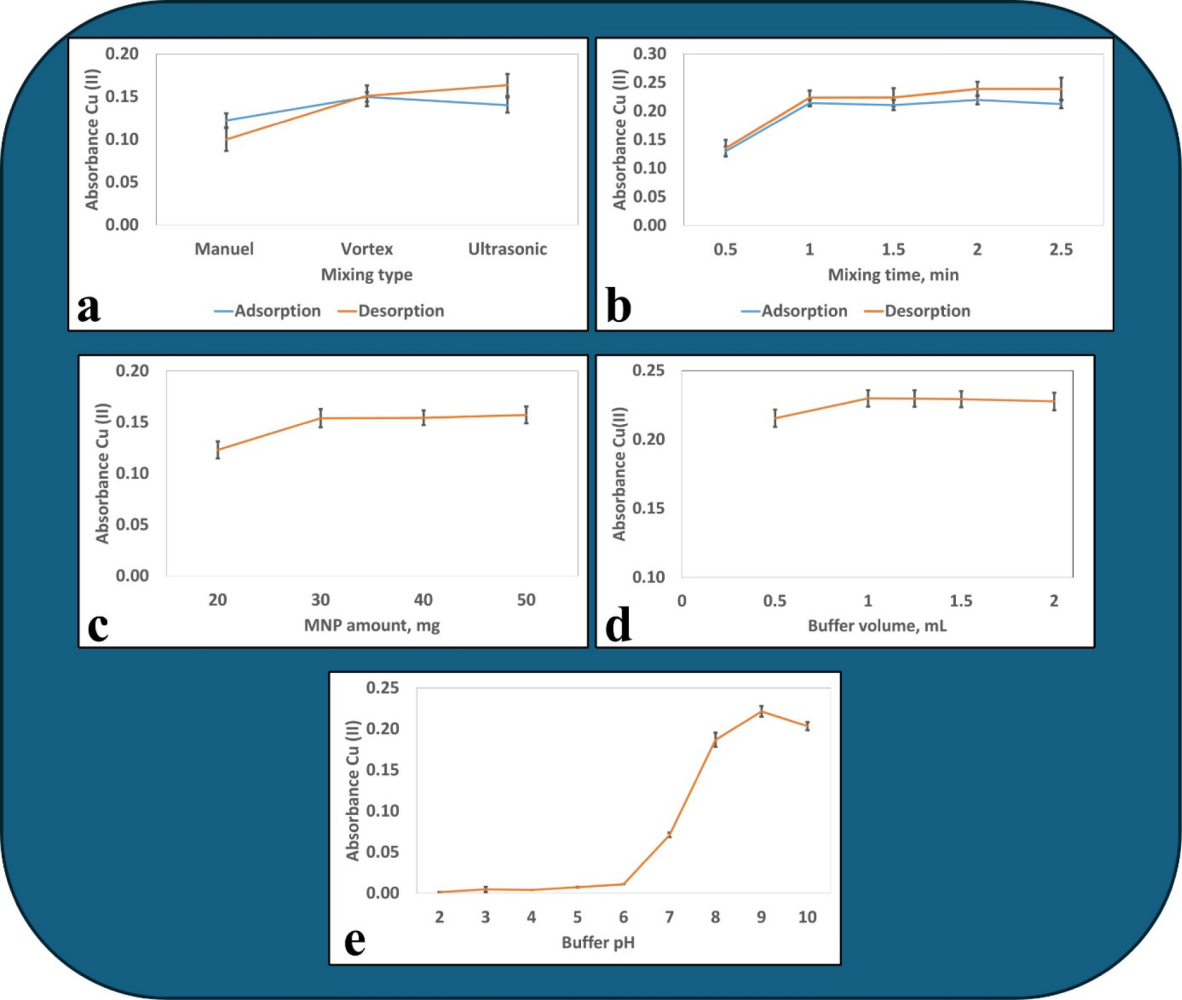
(a) Optimization of the mixing type for adsorption and desorption (Cu^2+^ concentration of the sample: 25.0 ng·mL^–1^, sample volume: 45 mL, pH = 9, amount of adsorbent: 20 mg, adsorption vortex time: 1 min, desorption vortex time: 0.5 min, volume of eluent: 0.3 μL). (b) Optimization of the mixing period for adsorption and desorption (Cu^2+^ concentration of the sample: 25.0 ng·mL^–1^, sample volume: 45 mL, pH = 9, amount of adsorbent: 20 mg, volume of eluent: 0.3 μL). (c) Optimization of the MNP quantity (Cu^2+^ concentration of sample: 25.0 ng·mL^–1^, sample volume: 45 mL, pH = 9, adsorption vortex time: 1 min, desorption vortex time: 1.0 min, volume of eluent: 0.3 μL). (d) Optimization buffer volume (Cu^2+^ concentration of the sample: 50.0 ng·mL^–1^, sample volume: 45 mL, amount of adsorbent: 20 mg, adsorption vortex time: 2 min, desorption vortex time: 0.5 min, volume of eluent: 0.3 μL). (e) Optimization of pH (Cu^2+^ concentration of the sample: 50.0 ng·mL^–1^, sample volume: 45 mL, amount of adsorbent: 20 mg, adsorption vortex time: 1 min, desorption vortex time: 0.5 min, volume of eluent: 0.3 μL); *N* = 3.

This study tested the pH range of 4–10 to determine the optimum pH for Cu(II) recovery (Figure 4e). It was observed that Cu(II) had lower absorbance values at low pH levels of SPE-FAAS due to the interference of hydronium ions hindered the interaction with adsorbent nanoparticles in an acidic environment.^25^ In the range of 7.0–10.0, the pH level was found to be more favorable for Cu(II) and adsorbent interaction, leading to increased recovery. At pH levels above 7.0, the adsorbent becomes negatively charged, enhancing metal ion adsorption.^26^ At pH 10.0, a slight decrease in the recovery was observed. The optimal pH of 9.0 was selected and used in the remaining experiments.

At an optimal pH of 9, both electrostatic interactions and coordination effects between Cu(II) ions and the adsorbent’s functional groups, such as hydroxyl and carboxyl groups, were maximized, as reported in previous studies.^27^ This pH also minimized competition from protons, enhancing adsorption efficiency and stabilizing Cu(II) ions through chelation, a mechanism.

To evaluate the effect of pH on the precipitation of Cu(II) ions independently of the adsorbent material, control experiments were conducted at pH 9 in the absence of an adsorbent. These experiments confirmed that no precipitation occurred in the solution without adsorbent at pH 9. These findings indicate that the observed results are primarily due to the interaction of the adsorbent material with Cu(II) ions rather than pH-induced precipitation alone. Also, Prabhakaran and Subramanian^28^ investigated the extraction of various metal ions using a chelating sorbent under high saline conditions and reported that adsorption efficiency was maximized in basic regions, specifically within the pH range of 5 to 10. Similarly, Soylak et al. conducted studies^29^ to determine the optimal pH for adsorption and performed experiments up to pH 10, further confirming the significance of basic pH conditions in enhancing adsorption performance. These findings align with the results of this study, in which pH 9 was identified as the optimal condition for Cu(II) ion adsorption.

Furthermore, we investigated various buffer solution volumes from 0.50 to 2.0 mL and found no significant difference. As a result, a buffer solution volume of 1.0 mL was selected and used in the remaining experiments. The optimization graph for the tested buffer solution volumes is presented in Figure 4d.

#### 3.2.2 Optimization of MNP Quantity

Optimization of the amount of adsorbent used in the MSPE extraction procedure is crucial to increase efficiency. When the adsorbent exceeds the optimum value, the elution volume required for desorption can reach saturation. Similarly, the recovery efficiency and enrichment factor may decrease if less than the optimum amount of adsorbent is used.^30^ For MNP amount optimization, triplicate trials (*N* = 3) with varying amounts (20, 30, 40 mg) of MNP were performed with a series of 45.0 mL of Cu(II)-containing solutions (Figure 4c). Using 30 mg of MNP yielded in a 1.5-fold increase in absorbance compared to 20 mg. While there was no significant difference between 30 and 40 mg, it was concluded that 30 mg was sufficient for recovery, and the optimum amount was determined as 30 mg of MNP.

#### 3.2.3 Optimization of Mixing Type and Period

Optimization of the mixing type and period is necessary to maximize the adsorption and desorption efficiencies in SPE processes. Efficient adsorption and desorption are directly related to the type and duration of mixing. For mixing time optimization, three methods—manual, vortex, and sonication—were tested for 1 min (Figure 4a). It was observed that vortexing for adsorption and sonication for desorption gave the highest absorbance. Periods of 0.5, 1.0, 1.5, 2.0, and 2.5 min were tested for adsorption and desorption, and 1.0 min was selected as the optimum value (Figure 4b).

Vortex mixing provides a turbulent flow that increases the contact between the adsorbent and the analyte. Thus, mass transfer rates and the distribution of adsorbent throughout the solution significantly improve the preconcentration efficiency.^31^ On the other hand, sonication increases desorption rates by applying high-frequency sound waves that create cavitation bubbles in the liquid medium. This phenomenon facilitates the release of adsorbed ions from the surface of the adsorbent and thus increases the recovery efficiency.^32^ Manual mixing could be less effective due to a lower energy input.

#### 3.2.4 Optimization of Eluent Type and Volume

This study aims to optimize the eluent type and volume in the desorption process of Cu(II) ions from hierarchical MnSb_2_O_6_@Fe_3_O_4_ (MNP) nanoparticles in the SPE process. Three eluents—HCl, HNO_3_, and CH_3_COOH—were evaluated for their efficiency in desorbing Cu(II) ions. HNO_3_ provided higher absorbance values than the other acids and was determined as the optimum eluent. The effectiveness of the eluent in SPE-FAAS is generally associated with its ability to protonate the adsorbent surface, displace adsorbed ions, and minimize interference in the flame path.^33^ HNO_3_, which is a strong oxidizing agent, can facilitate desorption by disrupting the interactions between Cu(II) ions and functional groups on the MNP surface. HNO_3_ is a strong acid that can provide the ion exchange necessary for effective desorption. Also, like organic acids, e.g., CH_3_COOH, it will not increase the background noise with organic structure residues in the flame pathway.

It is also essential to determine the optimum volume of eluent that minimizes waste and costs and ensures maximum recovery. Varying volumes of HNO_3_ (0.3, 0.4, 0.5, and 0.6 mL) were tested, and it was observed that an optimum of 0.3 mL HNO_3_ was sufficient to achieve the highest absorbance. Increasing the eluent volume leads to dilution of the analyte and a decreased concentration of recovered ions. Using smaller eluent volumes can increase the enrichment factor and lead to lower detection limits.

### 3.3 Analytical Figure of Merits

After optimizing the essential parameters, the method’s analytical performance was determined. The performance indicators calculated within the scope of the SPE-FAAS method of Cu(II) ions are limit of detection (LOD), limit of quantification (LOQ), coefficient of determination (*R*^2^), relative standard deviation (RSD %), preconcentration factor (PF), enhancement factor (EF), and linear working range (LR).^34^

The standard deviation was calculated from the absorbance values of 10 blank solutions and used for LOD and LOQ calculation. EF was calculated as the ratio of the slope of the calibration line obtained after the method to the slope of the FAAS calibration line. In addition, PF was calculated as the ratio of the final solution concentration to the initial solution concentration. The LOD and LOQ were determined to be 2.1 and 6.9 ng·mL^–1^, respectively, in the operating range of 10.0–200 ng·mL^–1^. The high linearity results, with an *R*^2^ value of 0.9991, underscore the accuracy of our method. The RSD was calculated as 4.2%. Method EF and PF values were calculated as 47.7 and 45.3, respectively.

Table 1 compares the proposed MnSb_2_O_6_@Fe_3_O_4_ MSPE-FAAS method with extraction studies for the Cu(II) ion reported in the literature and highlights its competitive performance.

**Table 1:** MNP-FAAS Method Performance and Literature Comparison

Method	Analyte	LOD^a^/LOQ^b^ ng·mL^–1^	LR^c^ ng·mL^–1^	%RSD	EF/PF^d^	Adsorption capacity mg·g^–1^	Real sample	Reference
MSPE-FAAS	Cu^2+^	2.1/6.9	10–200	4.2	47.7/45.3	15.2	wastewater,tap water,apple tea	this study
MWCNTs impregnated with D2EHPA-TOPO^e^	Cu^2+^	50.0/-	10–55	<10	25(EF)	4.90	electroplating wastewater	(35)
	Ni^2+^	40.0/-				4.78		
MNPs/SiO_2_-EDTA/ ICP-OES	Cu^2+^	0.39/1.3	0.1–200	2.4	150(EF)	36.9	ERM-CA713 (wastewater) ICP multielement standard	(36)
	Zn^2+^	0.12/0.39	0.1–200	0.18		30.9		
	Cd^2+^	0.06/0.21	0.1–100	1.49		59.5		
	Cr^3+^	0.15/0.5	0.1–100	1.93		34.32		
	Pb^2+^	0.76/2.51	0.1–100	3.10		108.8		
melon-peel biochar/CoFe_2_O_4_	Cu^2+^	0.41/-	-	2.34	50 (PF)	106.4	sea and stream waters pepper, black cabbage, eggplant, and tomato samples	(37)
	Cd^2+^	1.82/-		4.19		65.4		
	Pb^2+^	3.16/-		3.10		188.7		
magnetic TiO_2_- mPs@S^f^-based SPE (Cu^2+^)	Cu^2+^	0.14/0.47	0.05–10	0.50	20 (PF)	37.6	tap water, lake water, and wastewater	(38)
	Mn^2+^	0.28/0.94		1.50		38.5		
	Ni^2+^	0.28/1.0		2.09		27.9		
polyelectrolyte multilayer-based MSPE-FAAS	Cu^2+^	0.23	1–30	2.1	95.7 (EF)	14.7	boiler water, tap water, well water, Thai fragrant rice, glutinous rice, rice from Northeast China	(39)
BTI^g^-loaded on Dowex optipore V-493-preconcentration-FAAS	Cu^2+^	1.14/-	0.14–2.01	<9	37 (PF)	-	Mineral water, snow water, basic dialysis solution, acidic dialysis solution, tap water, walnut, black tea, chickpea	(40)
	Fe^3+^	2.01/-						
	Zn^2+^	0.14/-						
XAD-16-modified DCPIMI^h^	Cu^2+^	1.9	0.01–0.34	2.1	35	70.6	quince, radish, lottus, eucalyptus, cowslip, fenel, menta	(41)
	Zn^2+^	1.5	0.01–0.3	2.3	39	64.3		
	Mn^2+^	2.6	0.02–0.31	3.0	27	60.1		

a Limit of detection.

b Limit of quantification.

c LR: Linear range.

d EF: Enhancement factor; PF: preconcentration factor.

e Multiwalled carbon nanotubes impregnated with di(2-ethylhexyl phosphoric acid) and tri-*n*-octyl phosphine oxide.

f Sulfide-modified magnetic titanium dioxide microparticles.

g *Bacillus thuringiensis* israelensis loaded on Dowex optipore V-493.

h 3-((2,6-dichlorophenyl)(1*H*-indol-3-yl)methyl)-1*H*-indole.

### 3.4 Effect of Interfering Ions

In this study, the effects of matrix ions and certain transition metals on the determination of Cu(II) in environmental samples were investigated in detail. The influence of potential interfering ions was evaluated by adding known concentrations of various ions to a solution containing Cu(II). The solution’s Cu(II) concentration was fixed at 300 μg L^–1^, while the interfering ions’ concentrations varied between 25 and 1000 mg·L^–1^. The tolerance limit for each interfering ion was determined^35^ as the maximum concentration that resulted in a deviation of less than ±4% in Cu(II) adsorption efficiency, ensuring the accuracy of the method even in the presence of potentially interfering ions. The results are expressed as the mean ± standard deviation calculated from three replicates of the 45 mL sample presented in Table 2.

**Table 2:** Tolerance Levels of Interfering Ions for the Adsorption of Cu(II) Using the MnSb_2_O_6_@Fe_3_O_4_ Adsorbent

Ion	Added as	Concentration (mg·L^–1^)	Recovery (%)
Co^2+^	CoCl_2_	50	96.2 ± 2.2
Ni^2+^	Ni(NO_3_)_2_	20	94.1 ± 1.8
Cr^3+^	Cr(NO_3_)_3_	50	95.3 ± 1.3
Mg^2+^	MgCl_2_	1000	96.2 ± 2.1
Ca^2+^	CaCl_2_	1000	98.4 ± 3.6
K^+^	KNO_3_	1000	97.2 ± 2.4
Na^+^	NaCl	1000	97.8 ± 2.6
PO_4_^3–^	KH_2_PO_4_	1000	98.3 ± 2.8
Cl^–^	NaCl	1000	98.5 ± 2.2
NO_3_^–^	KNO_3_	1000	97.8 ± 2.8
SO_4_^2–^	Na_2_SO_4_	1000	98.5 ± 3.4

The obtained data indicated that the MnSb_2_O_6_@Fe_3_O_4_ adsorbent did not adsorb alkaline and alkaline earth metals commonly found in drinking water. This behavior can be attributed to the inability of these ions to form chelates and their weak interactions with the adsorbent surface under the experimental conditions. In contrast, heavy metal ions such as Ni^2+^, Cr^3+^, and Co^2+^ exhibited interactions with the adsorbent surface, leading to interference in the determination of Cu(II). At higher concentrations (400 mg·L^–1^) of Ni^2+^, Cr^3+^, and Co^2+^, interference with the preconcentration of Cu(II) was observed. This interference may arise from the partial occupation of the adsorbent’s active sites by these ions, which could slightly influence the efficiency of Cu(II) binding at elevated concentrations of interfering ions. These findings confirm that the MnSb_2_O_6_@Fe_3_O_4_ adsorbent demonstrated a high selectivity toward Cu(II) ions. This selectivity can be attributed to the strong interactions between Cu(II) ions and the active binding sites on the adsorbent surface.

### 3.5 Real Samples’ Application

To determine selectivity, comprehensive method validation was carried out by incorporating real sample applications into the MSPE–FAAS technique alongside interference studies. The presence of organic matter, salts, and other metal ions in real samples can cause interferences that complicate and affect the measurement accuracy. Various compounds in wastewater, such as heavy metals, nutrients, pathogens, drugs and personal care products, and organic pollutants, can affect the accuracy of the analysis through interference.^42^ Tap water is usually treated to remove harmful substances but may contain various constituents, including chlorine and chlorination byproducts, fluoride, minerals, microbial contaminants, and heavy metals. Apple tea may include polyphenolic compounds, amino acids, vitamins and minerals, and volatile compounds.^43^

The MSPE-FAAS method developed in this study was applied to various water (wastewater and tap water) and apple tea samples (*N* = 3). No signal was obtained in the method applied to blank samples, and the matrix-matching calibration method was applied to demonstrate the recovery performance. The MSPE-FAAS method was used for Cu(II) solutions prepared at various concentrations (20, 50, and 100 ng·mL^–1^; Table 3). The linear calibration lines, calculated recovery rates, and associated standard deviations are given in Table 3. According to the observed recovery results of 93 to 102% in this table, the proposed method is sufficient in accuracy and precision.

**Table 3:** Recovery Results of Cu(II) in Wastewater, Tap Water, and Apple Tea Samples Using MSPE–FAAS

Sample matrix	Cu(II) spiked concentration (ng·mL^–1^)	Recovery (%)	Standard Deviation ±
wastewater	20	93.1	± 5.2
	50	93.3	± 6.0
	100	94.4	± 4.5
tap water	20	95.1	± 4.8
	50	99.4	± 5.3
	100	101.3	± 4.7
apple tea	20	94.3	± 5.0
	50	97.1	± 5.8
	100	102.2	± 4.6

## 4 Conclusion

This study successfully synthesized and characterized hierarchical MnSb_2_O_6_@Fe_3_O_4_ magnetic nanoparticles, demonstrating their high efficiency as adsorbents for the preconcentration of Cu(II) ions using magnetic solid-phase extraction coupled with the flame atomic absorption spectroscopy (MSPE–FAAS) technique. The synthesized nanoparticles were thoroughly characterized using scanning electron microscopy (SEM) to examine the surface morphology and hierarchical structure and using energy-dispersive X-ray spectroscopy (EDX) to confirm the elemental composition. Fourier-transform infrared spectroscopy (FTIR) was employed to identify the functional groups present on the nanoparticle surface, while X-ray diffraction (XRD) analysis revealed the crystalline structure and confirmed the successful incorporation of the MnSb_2_O_6_ and Fe_3_O_4_ phases.

The MSPE-FAAS method’s sensitivity and precision were significantly improved by systematically optimizing critical parameters, including solution pH, quantity of adsorbent quantity, mixing technique, and eluent type and volume. These optimizations enhanced the adsorption and desorption efficiencies and minimized matrix interferences, ensuring reliable and reproducible results. The MSPE–FAAS method exhibited excellent analytical performance, achieving a detection limit of 2.1 ng·mL^–1^, representing a substantial enhancement compared to stand-alone FAAS. Additionally, the adsorption capacity of MnSb_2_O_6_@Fe_3_O_4_ for Cu(II) ions was determined, providing further evidence of the material’s high efficiency with a calculated capacity of 15.2 mg·g^–1^.

High recovery rates ranging from 93% to 102% were consistently achieved across complex sample matrices, including wastewater, tap water, and apple tea infusions, demonstrating the method’s high selectivity for Cu(II) ions. The study further investigated the tolerance levels of interfering ions, revealing that the developed method effectively resisted common matrix interferences, such as high concentrations of Na^+^, K^+^, Mg^2+^, and Cl^–^ ions, without compromising the adsorption efficiency. This exceptional selectivity highlights the robustness and reliability of the method in accurately quantifying Cu(II) ions even in challenging sample environments.

In conclusion, the MSPE–FAAS method demonstrated high reliability, reproducibility, and accuracy in quantifying trace levels of Cu(II) ions, even in the presence of challenging matrix interferences. These results highlight the method’s potential as a cost-effective, user-friendly, and environmentally sustainable solution for monitoring heavy metal contaminants in both environmental and food samples.

## Data Availability

The data underlying this study are not publicly available as they are part of an ongoing research project. However, the data are available from the corresponding author upon reasonable request.

## References

[ref1] aOdjegba V. J.; Fasidi I. O. Effects of heavy metals on some proximate composition of *Eichhornia crassipes*. J. Appl. Sci. Environ. Manage. 2006, 10 (1), 83–87. 10.4314/jasem.v10i1.17309.

[ref2] Jogdand O.; Bandela N. N.; Kaushik G.; Chel A. Determination of Select Heavy Metals in Air Samples from Aurangabad City. Handbook of Environmental Materials Management; Springer International Publishing, 2018; pp 1–10.

[ref3] aKüpper H.; Andresen E. Mechanisms of metal toxicity in plants. Metallomics 2016, 8 (3), 269–285. 10.1039/C5MT00244C. 26837424

[ref4] aOsorio-González C. S.; Hegde K.; Brar S. K.; Delgado-Cano B.; Gómez-Falcón N.; Avalos-Ramírez A. Advances in protein/enzyme-based biosensors for the detection of metal contaminants in the environment. In Tools, techniques and protocols for monitoring environmental contaminants; Elsevier, 2019; pp. 245–261.

[ref5] aCate D.; Volckens J.; Henry C. Personal exposure assessment to particulate metals using a paper-based analytical device. In Microfluidics, BioMEMS, and Medical Microsystems XI; SPIE, 2013; Vol. 8615, pp. 198–203.

[ref6] Jiang Q.; Liu Q.; Chen Q.; Zhao W.; Xiang G.; He L.; Jiang X.; Zhang S. Dicationic Polymeric Ionic-liquid-based Magnetic Material as an Adsorbent for the Magnetic Solid-phase Extraction of Organophosphate Pesticides and Polycyclic Aromatic Hydrocarbons. J. Sep. Sci. 2016, 39 (16), 3221–3229. 10.1002/jssc.201600267. 27357486

[ref7] aLouhi A.; Hammadi A.; Achouri M. Determination of some heavy metal pollutants in sediments of the Seybouse River in Annaba, Algeria. Air, Soil Water Res. 2012, 5, ASWR. S10081 10.4137/ASWR.S10081.

[ref8] Tülüce H. Â. Hegel’s Aesthetic Approach in the Concept and Image Specific. Din Bilim 2021, 4 (1), 6–16. 10.47145/dinbil.913347.

[ref9] Buldini P. L.; Cavalli S.; Mevoli A.; Sharma J. L. Ion chromatographic and voltammetric determination of heavy and transition metals in honey. Food Chem. 2001, 73 (4), 487–495. 10.1016/S0308-8146(01)00132-7.

[ref10] Arvand M. P.; Moghimi A.; Salehi N. A Novel Removal of Ni2+ Ions From Water Solutions Using Dispersive Solid-Phase Extraction Method With Nano Fe3O4/Chitosan-Acrylamide Hydrogel. Environ. Monit. Assess. 2024, 196 (2), 136 10.1007/s10661-023-12149-x. 38200248

[ref11] aMohammadi S. Z.; Shamspur T.; Karimi M.; Naroui E. Preconcentration of Trace Amounts of Pb(II) Ions Without Any Chelating Agent by Using Magnetic Iron Oxide Nanoparticles Prior to ETAAS Determination. Sci. World J. 2012, 2012, 1–6. 10.1100/2012/640437. PMC3353310 22649300

[ref12] Sharifi A.; Hallaj R.; Bahar S. Preconcentration of Pb(II) by Magnetic Metal-Organic Frameworks and Analysis Using Graphite Furnace Atomic Absorption Spectroscopy. J. Anal. Methods Chem. 2023, 2023, 1–10. 10.1155/2023/5424221. PMC9873434 36703710

[ref13] Bahramnezhad B.; Ghazanfari D.; Sheikhhosseini E.; Akhgar M. R.; Ahmadi S. A. MnSb2O6-chitosan nanocomposite: An efficient catalyst for the synthesis of coumarins via Pechmann reaction. J. Heterocycl. Chem. 2020, 57 (1), 173–181. 10.1002/jhet.3763.

[ref14] aYang C.; Pang Y.; Han Y.; Zhan X.; Wang H.; Liu J.; Gao R.; Liu H.; Shi H. Removal of trace concentration Sb (V) in textile wastewater by Mn-doped Fe3O4: The mechanisms of Mn affect adsorption performance. Microporous Mesoporous Mater. 2022, 343, 112150 10.1016/j.micromeso.2022.112150.

[ref15] Hayashi Y.; Matsuda R.; Maitani T.; Imai K.; Nishimura W.; Ito K.; Maeda M. Precision, Limit of Detection and Range of Quantitation in Competitive ELISA. Anal. Chem. 2004, 76 (5), 1295–1301. 10.1021/ac0302859. 14987084

[ref16] Xu F.; Cheng G.; Song S.; Wei Y.; Chen R. Insights into promoted adsorption capability of layered BiOCl nanostructures decorated with TiO2 nanoparticles. ACS Sustainable Chem. Eng. 2016, 4 (12), 7013–7022. 10.1021/acssuschemeng.6b01920.

[ref17] Djaja N. F.; Montja D. A.; Saleh R. The Effect of Co Incorporation Into ZnO Nanoparticles. Adv. Mater. Physics Chem. 2013, 03 (1), 33–41. 10.4236/ampc.2013.31006.

[ref18] aMalik L. A.; Bashir A.; Ahmad N.; Qureashi A.; Pandith A. H. Exploring Metal Ion Adsorption and Antifungal Properties of Carbon-Coated Magnetite Composite. Chemistryselect 2020, 5 (11), 3208–3216. 10.1002/slct.201904830.

[ref19] Liu Z.; Xing Y.; Fang S.; Qu X.; Wu D.; Zhang A.; Xu B. Low Temperature Self-Assembled Synthesis of Hexagonal Plate-ShapeMn_3_O_4_3D Hierarchical Architectures and Their Application in Electrochemical Capacitors. RSC Adv. 2015, 5 (68), 54867–54872. 10.1039/C5RA08697C.

[ref20] Kreider M. E.; Gunasooriya G. T. K. K.; Liu Y.; Zeledón J. A. Z.; Valle E.; Zhou C.; Montoya J. H.; Gallo A.; Sinclair R.; Nørskov J. K.; Stevens M. B.; Jaramillo T. F. Strategies for Modulating the Catalytic Activity and Selectivity of Manganese Antimonates for the Oxygen Reduction Reaction. ACS Catal. 2022, 12 (17), 10826–10840. 10.1021/acscatal.2c01764.

[ref21] Darwish A.; Ghoniem A.; Hassaan M. Y.; Shehata O. E-S.; Turky G. Synthesis and Characterization of Polyaniline/Mn3O4/Reduced Graphene Oxide Nanocomposite. Egypt. J. Chem. 2019, 62 (1), 251–265. 10.21608/ejchem.2019.13194.1821.

[ref22] Mehdi Z. S.; Alshamkhawy S. A. R. A. A Univariate Optimization Strategy for Pre-Concentration of Cobalt(II) in Various Matrixes by a DLLME Before Analysis Using FAAS. Indones. J. Chem. 2024, 24 (2), 403 10.22146/ijc.88218.

[ref23] Wang Q.; Yin C.-R.; Xu L. Optimization of Hydrophilic Interaction LC by Univariate and Multivariate Methods and Its Combination With Salting-out Liquid–liquid Extraction for the Determination of Antihypertensive Drugs in the Environmental Waters. J. Sep. Sci. 2013, 36 (6), 1007–1014. 10.1002/jssc.201200985. 23450627

[ref24] Huang J.; Liu X.; Thormann E. Surface forces between highly charged cationic polyelectrolytes adsorbed to silica: how control of pH and the adsorbed amount determines the net surface charge. Langmuir 2018, 34 (25), 7264–7271. 10.1021/acs.langmuir.8b00909. 29864283

[ref25] Fubara A.; Uche C.; Nwoko C.; Tony-Njoku R.; Ojiaku A.; Edo F. Assessment of the effectiveness of water hyacinth (E. crassipes) in the biosorption of heavy metals from Aluminium extruding company effluents. J. Appl. Sci. Environ. Manage. 2022, 26 (1), 37–46. 10.4314/jasem.v26i1.6.

[ref26] Ma L.; Wei Q.; Chen Y.; Song Q.; Sun C.; Wang Z.; Wu G. Removal of cadmium from aqueous solutions using industrial coal fly ash-nZVI. R. Soc. Open Sci. 2018, 5 (2), 171051 10.1098/rsos.171051. 29515830 PMC5830719

[ref27] Saçmaci Ş.; Kartal S.; Sacmaci M. Determination of Cr (III), Fe (III), Ni (II), Pb (II) and Zn (II) ions by FAAS in environmental samples after separation and preconcentration by solvent extraction using a triketone reagent. Fresenius Environ. Bull. 2012, 21 (6), 1563–1570.

[ref28] Prabhakaran D.; Subramanian M. S. A new chelating sorbent for metal ion extraction under high saline conditions. Talanta 2003, 59 (6), 1227–1236. 10.1016/S0039-9140(03)00030-4. 18969013

[ref29] Duran C.; Gundogdu A.; Bulut V. N.; Soylak M.; Elci L.; Sentürk H. B.; Tüfekci M. Solid-phase extraction of Mn (II), Co (II), Ni (II), Cu (II), Cd (II) and Pb (II) ions from environmental samples by flame atomic absorption spectrometry (FAAS). J. Hazard. Mater. 2007, 146 (1–2), 347–355. 10.1016/j.jhazmat.2006.12.029. 17223260

[ref30] Zhang X.; Shi X.; Ma L.; Pang X.; Li L. Preparation of Chitosan Stacking Membranes for Adsorption of Copper Ions. Polymers 2019, 11 (9), 1463 10.3390/polym11091463. 31500181 PMC6780952

[ref31] Lima A. F.; Richter E. M.; Muñoz R. A. A. Alternative Analytical Method for Metal Determination in Inorganic Fertilizers Based on Ultrasound-Assisted Extraction. J. Braz. Chem. Soc. 2011, 22 (8), 1519–1524. 10.1590/S0103-50532011000800016.

[ref32] aAkpomie K. G.; Conradie J. Biogenic and chemically synthesized Solanum tuberosum peel–silver nanoparticle hybrid for the ultrasonic aided adsorption of bromophenol blue dye. Sci. Rep. 2020, 10 (1), 17094 10.1038/s41598-020-74254-y. 33051565 PMC7555862

[ref33] Na J.; Lim S. Effect of Surface Oxidation on the Material Loss of InGaAs in Acidic Solutions. Solid State Phenom. 2021, 314, 89–94. 10.4028/www.scientific.net/SSP.314.89.

[ref34] Oshaghi S. Nano-sized magnetic molecularly imprinted polymer solid-phase microextraction for highly selective recognition and enrichment of sulfamethoxazole from spiked water samples. J. Chromatogr. A 2024, 1729, 465016 10.1016/j.chroma.2024.465016. 38852266

[ref35] Vellaichamy S.; Palanivelu K. Preconcentration and separation of copper, nickel and zinc in aqueous samples by flame atomic absorption spectrometry after column solid-phase extraction onto MWCNTs impregnated with D2EHPA-TOPO mixture. J. Hazard. Mater. 2011, 185 (2–3), 1131–1139. 10.1016/j.jhazmat.2010.10.023. 21041024

[ref36] Kobylinska N.; Kostenko L.; Khainakov S.; Garcia-Granda S. Advanced core-shell EDTA-functionalized magnetite nanoparticles for rapid and efficient magnetic solid phase extraction of heavy metals from water samples prior to the multi-element determination by ICP-OES. Microchim. Acta 2020, 187 (5), 289 10.1007/s00604-020-04231-9. 32335725

[ref37] Ozdes D.; Duran C. Preparation of melon peel biochar/CoFe 2 O 4 as a new adsorbent for the separation and preconcentration of Cu (II), Cd (II), and Pb (II) ions by solid-phase extraction in water and vegetable samples. Environ. Monit. Assess. 2021, 193 (10), 642 10.1007/s10661-021-09389-0. 34508274

[ref38] Diriba F.; Amde M.; Teju E. Sulfide-modified magnetic titanium dioxide microparticles for the adsorption and solid-phase extraction of toxic metals in aqueous samples. J. Dispersion Sci. Technol. 2023, 1–14. 10.1080/01932691.2023.2298872.

[ref39] Xiang G.; Ma Y.; Jiang X.; Mao P. Polyelectrolyte multilayers on magnetic silica as a new sorbent for the separation of trace copper in food samples and determination by flame atomic absorption spectrometry. Talanta 2014, 130, 192–197. 10.1016/j.talanta.2014.07.003. 25159398

[ref40] Tuzen M.; Melek E.; Soylak M. Solid-phase extraction of copper, iron and zinc ions on Bacillus thuringiensis israelensis loaded on Dowex optipore V-493. J. Hazard. Mater. 2008, 159 (2–3), 335–341. 10.1016/j.jhazmat.2008.02.021. 18359563

[ref41] Ghaedi M.; Niknam K.; Taheri K.; Hossainian H.; Soylak M. Flame atomic absorption spectrometric determination of copper, zinc and manganese after solid-phase extraction using 2, 6-dichlorophenyl-3, 3-bis (indolyl) methane loaded on Amberlite XAD-16. Food Chem. Toxicol. 2010, 48 (3), 891–897. 10.1016/j.fct.2009.12.029. 20060028

[ref42] Andreeva S. Improving the efficiency of technological processes for the treatment of highly concentrated wastewater to ensure environmental safety. In E3S Web of Conferences; EDP Sciences, 2021; p 06007.

[ref43] Liang Y.-R.; Lu J.-L.; Zhang L. Comparative Study of Cream in Infusions of Black Tea and Green Tea [Camellia Sinensis (L.) O. Kuntze]. Int. J. Food Sci. Technol. 2002, 37 (6), 627–634. 10.1046/j.1365-2621.2002.00589.x.

